# Assessing the cortical microstructure in contralesional sensorimotor areas after stroke

**DOI:** 10.1093/braincomms/fcae115

**Published:** 2024-05-28

**Authors:** Paweł P Wróbel, Stephanie Guder, Jan F Feldheim, Jose A Graterol Pérez, Benedikt M Frey, Chi-un Choe, Marlene Bönstrup, Bastian Cheng, Yogesh Rathi, Ofer Pasternak, Götz Thomalla, Christian Gerloff, Martha E Shenton, Robert Schulz

**Affiliations:** Department of Neurology, University Medical Center Hamburg-Eppendorf, 20246 Hamburg, Germany; Department of Neurology, University Medical Center Hamburg-Eppendorf, 20246 Hamburg, Germany; Department of Neurology, University Medical Center Hamburg-Eppendorf, 20246 Hamburg, Germany; Department of Neurology, University Medical Center Hamburg-Eppendorf, 20246 Hamburg, Germany; Department of Neurology, University Medical Center Hamburg-Eppendorf, 20246 Hamburg, Germany; Department of Neurology, University Medical Center Hamburg-Eppendorf, 20246 Hamburg, Germany; Department of Neurology, University Medical Center Hamburg-Eppendorf, 20246 Hamburg, Germany; Department of Neurology, University Medical Center, 04103 Leipzig, Germany; Department of Neurology, University Medical Center Hamburg-Eppendorf, 20246 Hamburg, Germany; Psychiatry Neuroimaging Laboratory, Brigham and Women’s Hospital, Harvard Medical School, Sommerville, MA 02145, USA; Department of Radiology, Brigham and Women’s Hospital, Harvard Medical School, Boston, MA 02115, USA; Psychiatry Neuroimaging Laboratory, Brigham and Women’s Hospital, Harvard Medical School, Sommerville, MA 02145, USA; Department of Radiology, Brigham and Women’s Hospital, Harvard Medical School, Boston, MA 02115, USA; Department of Neurology, University Medical Center Hamburg-Eppendorf, 20246 Hamburg, Germany; Department of Neurology, University Medical Center Hamburg-Eppendorf, 20246 Hamburg, Germany; Psychiatry Neuroimaging Laboratory, Brigham and Women’s Hospital, Harvard Medical School, Sommerville, MA 02145, USA; Department of Radiology, Brigham and Women’s Hospital, Harvard Medical School, Boston, MA 02115, USA; Department of Neurology, University Medical Center Hamburg-Eppendorf, 20246 Hamburg, Germany

**Keywords:** DWI, cortical, plasticity, unaffected, hemisphere

## Abstract

Cortical thickness analyses have provided valuable insights into changes in cortical brain structure after stroke and their association with recovery. Across studies though, relationships between cortical structure and function show inconsistent results. Recent developments in diffusion-weighted imaging of the cortex have paved the way to uncover hidden aspects of stroke-related alterations in cortical microstructure, going beyond cortical thickness as a surrogate for cortical macrostructure. Animal data obtained in rats and monkeys have evidenced that contralesional motor areas undergo degenerative alterations in their microstructure which are accompanied by compensatory changes as well. We hypothesized that cortical diffusion imaging can detect similar changes in human stroke survivors. We re-analysed clinical and imaging data of 42 well-recovered chronic stroke patients from two independent cohorts (mean age 64 years, 4 left-handed, 71% male, 16 right-sided strokes) and 33 healthy controls of similar age and gender. Cortical fractional anisotropy, axial diffusivity, radial diffusivity and cortical thickness values were obtained for six key sensorimotor areas of the contralesional hemisphere. The regions included the primary motor cortex, dorsal and ventral premotor cortex, supplementary and pre-supplementary motor areas and primary somatosensory cortex. Linear models were estimated for group comparisons between patients and controls and for correlations between cortical fractional anisotropy, axial diffusivity, radial diffusivity and cortical thickness and clinical scores. Against our hypothesis, we did not find any significant alterations in contralesional cortical microstructure after stroke. Likewise, we did not detect any correlations between cortical microstructure and behavioural scores. Future analyses are warranted to investigate whether such alterations might occur in different populations, e.g. in later stages of recovery, in more severely impaired patients, or only in the ipsilesional hemisphere in patients with specific lesion patterns.

## Teaser

Wrobel *et al*. report on cortical microstructural properties of contralesional sensorimotor areas in chronic stroke patients. Against their hypothesis, they could not detect any difference to healthy participants, nor could they detect any structure–behaviour associations. However, further analyses in different cohorts regarding time after stroke or symptom burden are warranted.

## Graphical Abstract

**Graphical Abstract:**
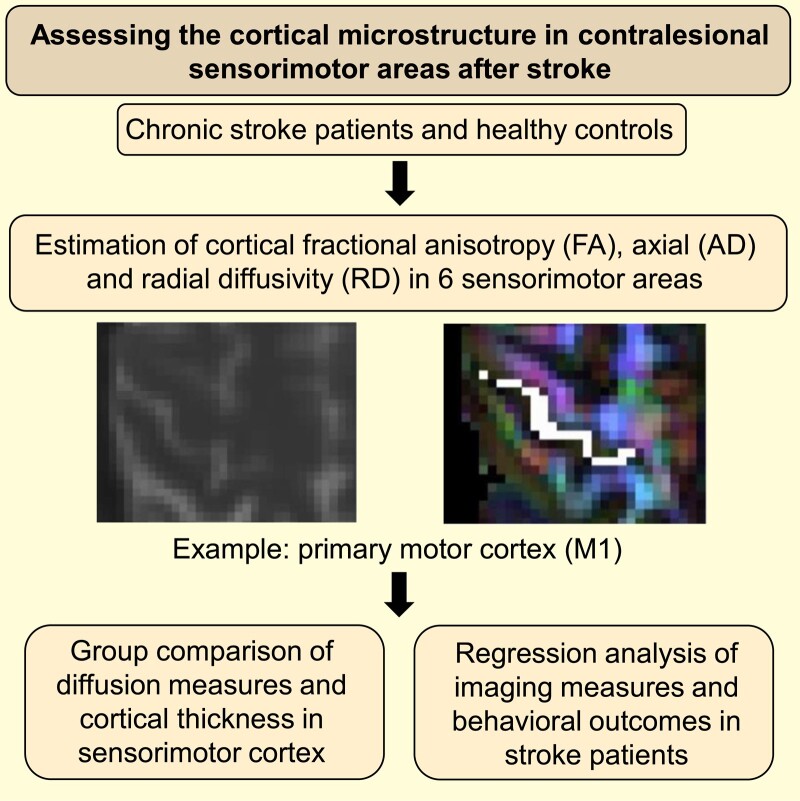

## Introduction

The assessment of macrostructural cortical changes after ischaemic stroke by means of volumetric or surface-based cortical thickness (CT) analyses has significantly enhanced our current understanding of how acute stroke lesions lead to widespread changes of brain structure and how these may influence recovery processes.^1^ Most studies have reported stroke-related cortical thinning in primary and secondary motor- and non-motor areas not only of the ipsilesional but also of the contralesional hemisphere.^2-9^ Some studies have also shown increases in CT or grey matter volume and argued that such findings might parallel neuroplastic brain changes, potentially promoting recovery after stroke.^10-13^ However, studies exhibit a large variability in such results for group comparisons and particularly for correlational analyses between CT and residual function as well.^1^ Recent developments have focused on layer-specific CT changes and their associations with clinical scores,^14^ which suggests that, in addition to CT as a surrogate for macrostructure, measures of cortical microstructure might carry important information of stroke-related changes in brain structure with the potential to improve our current recovery models.

Over the last 25 years, diffusion-weighted imaging (DWI) has provided invaluable insights into changes of brain networks and microstructure after stroke.^15^ Diffusion properties such as fractional anisotropy (FA), amongst others, have been evolved as surrogate parameters for white matter integrity. Technical limitations, such as free water (FW) contamination of DWI signals due to partial volume effects, have largely limited the application of DWI to cerebral white matter or deep subcortical grey matter.^16-20^ Recently developed methods such as FW correction^21^ or multi-shell DWI^16^ have extended the possibilities for cortical DWI. The cortex is not only organized in different layers, but also consists of capillaries, a variety of glia cells and neurones, which in turn display an intricate shape with the soma, axons, dendrites and synapses.^22^ Diffusion tensor imaging (DTI) has demonstrated that the main diffusion direction, i.e. the orientation of the diffusion tensor, was oriented perpendicularly to the pial surface and highly corresponded with tensor information derived from brain histology.^23^ Neuronal cell bodies, as well as the axon and apical dendrites of the neurones, were found to primarily contribute to this orientation. Together with other structures aligned orthogonally to the neurones and parallel to the pial surface, these were also reported to modulate anisotropy of the cortex. Dendritic arborization has been inversely correlated with the amount of anisotropy.^24, 25^ Dendrogenesis and synaptogenesis have been shown to reduce cortical FA over time during maturation.^26, 27^ In addition to FA, measures of regional variability of diffusion properties in the cortex have been related to clinical scores during aging processes.^28^ These findings illustrate that cortical DWI might serve as an innovative tool to uncover yet hidden aspects of cortical microstructural changes and their relevance for recovery after stroke. Examples of such microstructural changes, e.g. in contralesional motor cortices, have been reported by studies in animal stroke models. For instance, rat and macaque monkey data have revealed neurodegenerative processes in contralesional primary motor and premotor areas which are accompanied by compensatory neuroplastic changes including dendritic growth.^29, 30^ Human functional imaging data have repeatedly shown an upregulation in brain activity of contralesional motor and premotor areas, particularly in more impaired patients.^31-33^ In search of a structural parallel for such alterations after stroke, we hypothesized that cortical DTI would be capable to evidence comparable cortical microstructural changes in human stroke survivors. This study sought to investigate this in a group of well-recovered chronic stroke patients. We re-analysed clinical and imaging data of patients with first-ever ischaemic stroke in the chronic stage of recovery, taken from two independent studies.^34, 35^ Following a region-of-interest approach, three different diffusion measures were calculated for six key sensorimotor brain regions obtained from the human motor atlas template (HMAT).^36^ Fractional anisotropy was obtained as a surrogate for cortical microstructural complexity,^26, 27^ and axial diffusivity (AD) and radial diffusivity (RD) were computed as surrogates for directed water diffusion along the cortical columns, probably representing the number of neurones or afferent axons, or along the structures parallel to the cortex, such as dendrites and interneurones, respectively. Cortical thickness was also estimated for these brain regions to compare microstructural finding with cortical macrostructure. The analyses were restricted to the contralesional hemisphere to exclude any direct lesion effects. Linear regression models were fit (i) to conduct group comparisons for cortical diffusion measures and CT between stroke patients and controls and (ii) to relate these measures to clinical scores of residual motor output.

## Methods

### Participants and clinical data

**Figure 1:**
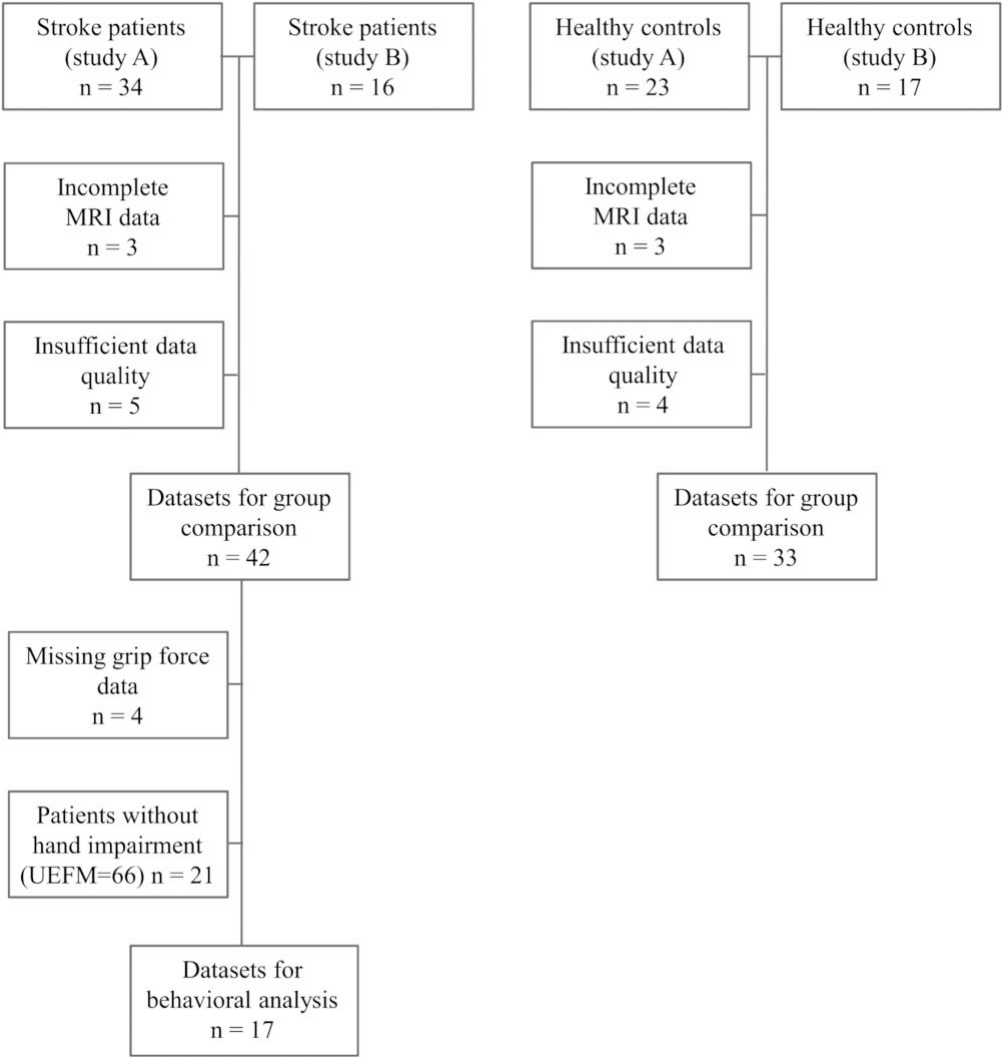
**Flowchart of participant inclusions and exclusions.** UEFM, upper extremity Fugl–Meyer.

Imaging and clinical data of chronic stroke patients and data of healthy controls of similar age and gender were re-analysed from two independent cohorts. Details of inclusion and exclusion criteria are given in the original reports.^34, 37^ In brief, patients had experienced a first-ever unilateral ischaemic stroke at least 6 months ago. A motor deficit of the upper limb was present, or it had been documented in the acute stage after stroke with subsequent improvement. Patients were at least 18 years old. Serious neurological or psychiatric comorbidities, contraindications against MRI, and evidence for a recurrent stroke were exclusion criteria. All participants gave informed written consent, compliant with the Declaration of Helsinki. The studies were approved by the ethics committee at the Physicians’ Chamber in Hamburg (PV3777 and PV5357). Figure 1 gives an overview of participant inclusion. The mean age of the stroke patients was 64 years, 71% were male, and 38 were right-handed. The median time after stroke was 12 months (range 7–88). Clinical testing included the National Institutes of Health Stroke Scale (NIHSS), the Upper Extremity Fugl–Meyer (UEFM) Assessment, grip strength (in kg, given as absolute and relative values: more affected/unaffected hand) and performance in the Nine-Hole-Peg (NHP) test (given as absolute values in pegs/sec and as relative values). For group comparison, 42 patients and 33 healthy participants were analysed. Analyses for structure–outcome correlations were conducted primarily in a subset of patients with persistent motor deficits, operationalized by the UEFM < 66. The rational for this approach was that previous studies have found that patients with complete or almost complete recovery were found to exhibit much weaker relationships between brain structure and outcome^38, 39^ and that structure–outcome relationships might be particularly driven by more impaired patients and might not generalize to patients with minor deficits.^40^ Table 1 summarizes the demographic and clinical data.

**Table 1:** Demographic and clinical data of the patients

ID	Age	T	Gender	Dom	Side	Localization	NIHSS	UEFM	Grip force (kg) AH | UH		NHP (pegs/s) AH | UH		Grip force (rel.)	NHP (rel.)
01	56	12	m	left	right	CI, BG	5	6	-	-	-	-	-	-
02	69	12	m	left	right	MCA-C	6	5	-	-	-	-	-	-
03	62	12	m	left	left	CI, BG	0	66	33.4	42.3	0.8	0.8	0.8	1.0
04	69	12	m	right	right	MCA-C	0	66	47.7	53.3	0.6	0.8	0.9	0.8
05	68	12	f	left	right	BS	0	66	23.2	21.8	0.8	0.9	1.1	0.9
06	71	12	m	left	right	CI	0	66	37.3	37.7	0.6	0.7	1.0	0.8
07	65	12	m	left	left	CI, BG	0	66	30.8	33.8	0.8	0.7	0.9	1.1
08	73	12	f	left	right	MCA-C	0	66	25.7	28.5	0.6	0.9	0.9	0.7
09	58	12	m	left	right	BS	2	58	23.7	45.0	0.7	0.9	0.5	0.7
10	73	12	f	left	left	CI, BG	6	6	7.9	-	-	-	-	-
11	56	12	f	left	right	MCA-C	0	66	32.7	36.0	0.9	1.0	0.9	0.9
12	72	12	f	left	left	BS	0	65	25.7	24.7	0.8	0.8	1.0	1.0
13	49	12	m	left	left	MCA-C	0	66	49.3	48.3	1.0	1.1	1.0	1.0
14	63	12	m	left	left	CI, BG	0	66	38.7	43.7	0.8	0.9	0.9	0.9
15	70	12	f	left	right	MCA-C	1	65	19.0	26.0	0.6	0.8	0.7	0.7
16	65	12	m	right	left	MCA-C	5	13	-	54.7	-	0.6	-	-
17	85	12	f	left	right	MCA-C	3	50	8.7	21.0	-	0.5	0.4	-
18	81	12	m	left	left	BS	0	66	41.0	40.0	0.5	0.5	1.0	1.0
19	44	12	m	right	left	MCA-C	1	66	37.7	45.7	0.9	1.0	0.8	0.8
20	78	12	f	left	left	CI, BG	0	66	26.3	24.0	0.9	0.9	1.1	1.0
21	47	12	f	left	right	MCA-C	2	64	18.7	23.7	0.5	0.9	0.8	0.6
22	81	12	m	left	left	MCA-C	3	63	24.7	28.0	0.4	0.5	0.9	0.8
23	76	12	m	left	left	BS	0	66	42.0	41.0	0.6	0.7	1.0	0.9
24	55	12	m	left	left	CI, BG	0	66	32.0	25.0	0.9	0.8	1.3	1.2
25	48	12	m	left	left	A-/MCA-C	2	66	53.3	48.0	0.8	0.5	1.1	1.3
26	66	12	m	right	right	MCA-C	2	66	54.6	52.7	0.6	0.9	1.0	0.7
27	47	12	m	left	right	CI, BG	2	44	7.6	34.0	-	0.8	0.2	-
28	77	28	m	left	left	CI, BG	3	60	36.3	40.3	0.4	0.6	0.9	0.6
29	78	20	f	left	left	CI, BG	0	66	19.3	24.0	0.8	0.8	0.8	1.0
30	74	20	m	left	left	CI	0	66	42.0	34.7	0.6	0.5	1.2	1.0
31	73	26	f	left	right	MCA-C	4	52	9.7	19.3	0.3	0.7	0.5	0.4
32	61	35	m	left	right	MCA-C	0	66	28.7	45.3	0.7	0.7	0.6	0.9
33	55	66	m	left	left	CI, BG	3	66	46.3	45.3	0.7	0.9	1.0	0.7
34	54	29	m	left	left	CI	2	59	17.3	42.0	0.6	0.7	0.4	0.8
35	61	75	m	left	left	CI, BG	3	47	31.0	42.7	0.5	0.9	0.7	0.6
36	76	80	m	left	left	CI, BG	2	50	24.3	31.7	0.4	0.8	0.8	0.6
37	61	88	m	left	left	PLIC	0	64	31.0	34.7	0.7	0.7	0.9	1.0
38	60	9	m	left	left	CI, BG	2	55	35.3	43.3	0.6	0.8	0.8	0.8
39	58	31	m	left	left	CI, BG	0	66	24.0	22.3	0.9	0.9	1.0	1.1
40	64	7	m	left	right	CI, BG	4	52	4.3	21.3	0.5	0.8	0.2	0.6
41	63	11	m	left	left	CI, BG	4	51	20.3	46.0	0.5	1.0	0.4	0.5
42	84	23	f	left	left	CI, BG	2	39	9.3	15.0	0.4	0.7	0.6	0.6

Mean age in years: stroke group—63.86 (SD: 10.86) and control group—66. (SD: 11.56), *P* = 0.279 (*t*-test) [median age: stroke group—65 years and healthy controls—69 years]. T: time after stroke onset in months (median: 12 months, range 7–88). Gender ratio (♂ [m]:♀[f]) stroke group 71:29% and control group 65:35%, *P* = 0.621 (Fisher’s exact test). Handedness ratio (r/l): stroke group—38:4 and control group—41:1, *P* = 0.315 (Fisher’s exact test). Median NIHSS in stroke patients: 1, median UEFM: 65, median relative grip force: 0.92, median NHP: 0.88.

Dom, dominant hemisphere; Side, affected hemisphere; CI, Capsula interna; BG, basal ganglia; MCA-C, A. cerebri media ischaemia with cortical and subcortical lesions; BS, brainstem; AH, affected hand; UH, unaffected hand; Rel, relative.

### Brain imaging—data acquisition, pre-processing and free water correction

Brain imaging was performed on a 3-tesla Siemens Skyra scanner (Siemens Medical Solutions, Erlangen, Germany) with a 32-channel head coil. T_1_-weighted imaging was based on a magnetization-prepared, rapid acquisition gradient-echo sequence (repetition time = 2.500 ms, echo time = 2.12 ms, 256 slices with a field of view = 240 × 192 mm and voxel size = 0.94 × 0.94 × 0.94 mm). Diffusion-weighted imaging data sets consisted of 75 slices with 64 non-collinear gradient directions with a *b*-value of 1500 s/mm² and one *b*_0_ image (echo time of 82 ms, repetition time of 10 000 ms and voxel size of 2 × 2 × 2 mm). Pre-processing of DWI datasets included eddy current correction using the FMRIB Software Library (FSL) (6.0.1)-based dwifslpreproc tool from MRtrix3 software (3.0.2) and correcting for phase-encoding distortion by applying data from registration of the *b*_0_ to the T_1_ image using the antsRegistration tool from the Advanced Normalization Tool (ANT) package.^41-43^ A custom-written MATLAB script (ran on version R2020a, The MathWorks, Natick, MA, USA) was used to perform FW correction following the approach of Pasternak *et al.*^21^ In brief, a bi-tensor model is calculated to predict the signal attenuation in the presence of FW contamination. The model includes two different compartments: the first compartment estimates the fractional volume of FW, which is modelled as an isotropic tensor with a fixed diffusivity, and the other compartment uses a diffusion tensor to model water molecule diffusion in the vicinity of tissue membranes, from which eigenvalue maps corrected for FW are calculated. T_1_-weighted images were processed using an ANT-based brain extraction tool and the *recon-all* tool from the FreeSurfer software (version 6.0.1).^44^

### Brain imaging—diffusion properties of key sensorimotor areas and CT

The HMAT was used to identify six cortical key areas of the human sensorimotor network of the contralesional hemisphere, which are the primary motor cortex (M1), the primary somatosensory cortex (S1), the ventral and dorsal premotor cortex (PMV and PMD) and the pre-supplementary (pre-SMA) and the supplementary motor area (SMA).^36^ The HMAT atlas labels in the Montreal Neurological Institute space were transformed non-linearly to the individual DWI space using the *flirt* and *fnirt* tools from FSL. The delineation of the final cortical labels was performed by the multiplication of each label with a binary cortex mask derived from the FreeSurfer-generated *wmparc* file by merging all cortical labels into a cortex mask using *fslmaths*. The HMAT labels were than multiplied with a binary mask of the label-specific lobe to avoid inclusion of voxels from other lobes. Regional mean CT of the six areas was estimated by registering the HMAT atlas data to the individual subject segmentation output using FreeSurfer’s *vol2surf* and finally collected via *mri_segstats*. The mean values for cortical FA, AD and RD for each of the six areas were derived from the diffusion tensor image volume consisting of the tensor eigenvalues using a custom-written MATLAB script (ran on version R2020a, The MathWorks, Natick, MA, USA) as previously described.^28^

### Statistical analysis

Statistics were performed using R software version 4.0.2. For group comparisons of cortical FA, AD, RD and CT, we estimated linear regression models with the cortical measures of interests as the dependent variable (DV) and GROUP (patients, controls) as the independent variable of interest. Separate models were calculated for all six regions of interest (ROIs), for cortical DTI measures (18 models) and CT values (six models). Target effects of the models were adjusted for the nuisance variables age, study, sex, lesion side (dominant or non-dominant hemisphere) and CT values in models for DTI measures. To account for the distribution of affected hemispheres in the patients (dominant or non-dominant), the assignment of hemispheres in controls was pseudorandomized into a pseudo-affected and a pseudo-unaffected hemisphere in line with previous studies. Going beyond our primary hypothesis and a region-wise approach, we also computed four linear mixed-effects models (one for each diffusion parameter and CT) for *post hoc* analyses, combining data of all six regions. Region was treated as an additional fixed effect; subject was treated as a random effect. For structure–outcome inference, linear regression models were fit for relative grip force and NHP performance, NIHSS and UEFM as DV. These analyses were conducted in a subset of patients with persistent motor deficits as operationalized by the UEFM < 66 (*n* = 17). Age, study and side of the lesion were included as nuisance variables to adjust the target effects, while the models for diffusion models were additionally adjusted for the CT. Statistical significance was assumed at *P* < 0.05. Results are presented uncorrected (*P*) and corrected for multiple comparisons using the false discovery rate (FDR) method (*P*_FDR_).^45^

## Results

### Cortical diffusion properties and thickness after stroke

Table 2 summarizes the results of the group comparison for regional FA, AD, RD, and CT values. Against our hypothesis, we did not find any significant group differences between stroke patients and healthy controls.

**Table 2:** Cortical diffusion properties and thickness after stroke

Parameter	Region	Controls (95% CI)	Stroke (95% CI)	*P*	*P*_FDR_
FA	M1	0.158 (0.150–0.165)	0.159 (0.153–0.166)	0.724	0.922
	PMD	0.143 (0.138–0.148)	0.145 (0.140–0.149)	0.543	0.922
	PMV	0.143 (0.138–0.148)	0.140 (0.136–0.145)	0.338	0.816
	Pre-SMA	0.147 (0.142–0.153)	0.155 (0.151–0.160)	0.020	0.240
	SMA	0.148 (0.143–0.153)	0.153 (0.148–0.158)	0.183	0.816
	S1	0.169 (0.159–0.179)	0.169 (0.159–0.178)	0.955	0.955
AD	M1	6.78 × 10^−4^ (6.69 × 10^−4^–6.86 × 10^−4^)	6.85 × 10^−4^ (6.77 × 10^−4^–6.93 × 10^−4^)	0.214	0.816
	PMD	6.68 × 10^−4^ (6.60 × 10^−4^–6.75 × 10^−4^)	6.70 × 10^−4^ (6.63 × 10^−4^–6.77 × 10^−4^)	0.575	0.922
	PMV	6.83 × 10^−4^ (6.80 × 10^−4^–6.87 × 10^−4^)	6.84 × 10^−4^ (6.80 × 10^−4^–6.87 × 10^−4^)	0.921	0.955
	Pre-SMA	6.76 × 10^−4^ (6.69 × 10^−4^–6.83 × 10^−4^)	6.81 × 10^−4^ (6.74 × 10^−4^–6.88 × 10^−4^)	0.348	0.816
	SMA	6.78 × 10^−4^ (6.71 × 10^−4^–6.86 × 10^−4^)	6.82 × 10^−4^ (6.75 × 10^−4^–6.89 × 10^−4^)	0.408	0.816
	S1	6.87 × 10^−4^ (6.77 × 10^−4^–6.96 × 10^−4^)	6.92 × 10^−4^ (6.83 × 10^−4^–7.01 × 10^−4^)	0.387	0.816
RD	M1	5.35 × 10^−4^ (5.27 × 10^−4^–5.42 × 10^−4^)	5.40 × 10^−4^ (5.33 × 10^−4^–5.47 × 10^−4^)	0.230	0.816
	PMD	5.37 × 10^−4^ (5.31 × 10^−4^–5.44 × 10^−4^)	5.38 × 10^−4^ (5.32 × 10^−4^–5.45 × 10^−4^)	0.842	0.955
	PMV	5.52 × 10^−4^ (5.49 × 10^−4^–5.55 × 10^−4^)	5.56 × 10^−4^ (5.53 × 10^−4^–5.58 × 10^−4^)	0.101	0.808
	Pre-SMA	5.40 × 10^−4^ (5.34 × 10^−4^–5.47 × 10^−4^)	5.39 × 10^−4^ (5.33 × 10^−4^–5.45 × 10^−4^)	0.730	0.922
	SMA	5.44 × 10^−4^ (5.38 × 10^−4^–5.50 × 10^−4^)	5.44 × 10^−4^ (5.37 × 10^−4^–5.49 × 10^−4^)	0.840	0.955
	S1	5.33 × 10^−4^ (5.26 × 10^−4^–5.39 × 10^−4^)	5.37 × 10^−4^ (5.31 × 10^−4^–5.43 × 10^−4^)	0.332	0.816
CT	M1	1.94 (1.89–2.00)	1.91 (1.86–1.96)	0.936	0.955
	PMD	2.33 (2.26–2.40)	2.29 (2.22–2.35)	0.674	0.922
	PMV	2.41 (2.35–2.48)	2.32 (2.26–2.39)	0.018	0.240
	Pre-SMA	2.67 (2.57–2.76)	2.63 (2.53–2.72)	0.324	0.816
	SMA	2.47 (2.38–2.57)	2.45 (2.33–2.58)	0.727	0.922
	S1	1.89 (1.84–1.93)	1.87 (1.83–1.92)	0.580	0.922

Estimated mean values [with 95% confidence intervals (CI)] are given for cortical FA, AD, RD, and CT for stroke patients (*n* = 42) and controls (*n* = 33). Level of significance is given for the main effect GROUP without (*P*) and with correction for 24 comparisons for FA, AD, RD, and CT each (*P*_FDR_).

### Structure–outcome relationships

Despite that group differences were not detected, we questioned whether cortical diffusion measures or CT within the sensorimotor areas tested might be associated with functional scores in the patients. However, there were no significant associations (all *P*_FDR_ > 0.1).

## Discussion

Previous animal data have suggested that contralesional motor and premotor areas show stroke-related changes in the cortical microstructure.^29, 30^ We hypothesized that cortical diffusion imaging would be capable to evidence comparable changes in human stroke survivors. However, the present data did not show any evidence for changes in cortical microstructure in six sensorimotor areas of the contralesional hemisphere.

Numerous studies have already addressed CT changes after stroke. They have been mostly reported for the ipsilesional hemisphere, and contralesional alterations have been shown to be less pronounced.^3, 4, 46^ However, their relationship with motor recovery remains under debate given positive^6, 11, 12, 14^ and negative^2, 4, 5, 9, 10^ study results. Compared with CT, studies addressing cortical microstructure after stroke are remarkably limited. One study used cortical diffusional kurtosis imaging and found that baseline values in postcentral, supramarginal and angular gyrus were significant predictors of gains in language treatment in 26 chronic stroke patients.^47^ However, one important limitation of this study was that a correction for FW was not included.^48^ The same limitation might apply to another small study in ten stroke patients.^16^ These patients underwent repeated MRI between 1 week and 6 months after stroke and exhibited an early increase in cortical FA in multiple temporo-frontal and motor areas gradually decreasing towards normality within 6 months. Thus, on the one hand, the present findings might argue against the presence of region-specific contralesional microstructural alterations after stroke, suggested by animal work.^29, 30^ On the other hand, obtained in relatively small cohorts, the data might simply indicate a reduced methodological sensitivity and would ask for much larger sample sizes for future, prospective investigations. As a first attempt, we added a *post hoc* linear mixed-effects analysis with repeated measures combining the diffusion/CT measures across all six regions of interest to enhance the statistical power. Four separate models were fitted to detect group differences for FA, AD, RD and CT. However, also these models did not detect any significant group effect (all *P* > 0.17).

There are several limitations to note. First, the present work was based on a relatively small group of well-recovered stroke patients. Therefore, the negative results might be explained by the reduced sensitivity of the method to detect subtle changes in the diffusion measures. Further, more severely impaired patients with larger lesion volumes might accentuate cortical alterations and give different results. Second, stroke locations were heterogeneous. This variability might directly translate into an increased variability in the distribution of secondary adaptive or maladaptive microstructural cortical alterations on the contralesional hemisphere. Third, most patients were at the end of the first year after stroke. This could indicate that more time after stroke is needed to allow for the detection of changes, and investigations in patients later after stroke might be reasonable. Fourth, the analyses were limited to the contralesional hemisphere to exclude direct lesion effects to the cortex. Ipsilesional cortices might exhibit more pronounced alterations, e.g. when analysed in specific subgroups of patients with isolated subcortical lesions or lesions which only affect the primary motor cortex and spare neighbouring premotor cortices.^49, 50^

## Data Availability

Data will be made available upon reasonable request.
