# Correction to: Assessing the cortical microstructure in contralesional sensorimotor areas after stroke

**DOI:** 10.1093/braincomms/fcae238

**Published:** 2024-07-19

This is a correction to: Paweł P Wróbel, Stephanie Guder, Jan F Feldheim, Jose A Graterol Pérez, Benedikt M Frey, Chi-un Choe, Marlene Bönstrup, Bastian Cheng, Yogesh Rathi, Ofer Pasternak, Götz Thomalla, Christian Gerloff, Martha E Shenton, Robert Schulz, Assessing the cortical microstructure in contralesional sensorimotor areas after stroke, *Brain Communications*, Volume 6, Issue 3, 2024, fcae115, https://doi.org/10.1093/braincomms/fcae115

In the originally published version of this paper the phrase “Retraction and replacement” was inadvertently included in the title. This error has been corrected.

